# Neural Regulation of Pancreatic Cancer: A Novel Target for Intervention

**DOI:** 10.3390/cancers7030838

**Published:** 2015-07-17

**Authors:** Aeson Chang, Corina Kim-Fuchs, Caroline P. Le, Frédéric Hollande, Erica K. Sloan

**Affiliations:** 1 Monash Institute of Pharmaceutical Sciences, Monash University, Parkville, Victoria 3052, Australia; E-Mails: aeson.chang@monash.edu (A.C.); corina.kim-fuchs@insel.ch (C.K.-F.); caroline.le@monash.edu (C.P.L.); frederic.hollande@unimelb.edu.au (F.H.); 2 Department of Visceral Surgery and Medicine, University Hospital Bern, Bern 3010, Switzerland; 3 Department of Pathology, University of Melbourne, Parkville 3010, Australia; 4 Cousins Center for PNI, UCLA Semel Institute, Jonsson Comprehensive Cancer Center, and UCLA AIDS Institute, University of California Los Angeles, Los Angeles, CA 90095, USA; 5 Peter MacCallum Cancer Centre, Division of Cancer Surgery, East Melbourne, Victoria 3002, Australia

**Keywords:** pancreatic cancer, beta-adrenergic, stress, beta-blockers, neural, metastasis

## Abstract

The tumor microenvironment is known to play a pivotal role in driving cancer progression and governing response to therapy. This is of significance in pancreatic cancer where the unique pancreatic tumor microenvironment, characterized by its pronounced desmoplasia and fibrosis, drives early stages of tumor progression and dissemination, and contributes to its associated low survival rates. Several molecular factors that regulate interactions between pancreatic tumors and their surrounding stroma are beginning to be identified. Yet broader physiological factors that influence these interactions remain unclear. Here, we discuss a series of preclinical and mechanistic studies that highlight the important role chronic stress plays as a physiological regulator of neural-tumor interactions in driving the progression of pancreatic cancer. These studies propose several approaches to target stress signaling via the β-adrenergic signaling pathway in order to slow pancreatic tumor growth and metastasis. They also provide evidence to support the use of β-blockers as a novel therapeutic intervention to complement current clinical strategies to improve cancer outcome in patients with pancreatic cancer.

## 1. Introduction

The tumor microenvironment (TME) is increasingly recognized for its crucial role in influencing cancer progression [1] and response to therapy [2,3]. The close spatial proximity between pancreatic cancer cells and the dense stromal cell population within the pancreas creates an environment that favors pancreatic cancer cell proliferation and dissemination, and impedes access of chemotherapeutic drugs [3,4,5,6]. These factors compound the issues of a disease that is frequently diagnosed late, resulting in an exceptionally poor five-year survival rate of 5% [7].

Similar to many other tumor types, the pancreatic TME contains many cellular and structural components such as tumor-associated macrophages and blood and lymphatic vasculature that contribute to the trajectory of pancreatic cancer progression. However, the pancreatic TME also contains unique features such as the presence of pancreatic stellate cells (PSCs), which contribute to the extensive desmoplastic reaction and fibrosis associated with pancreatic cancer [8,9], and a dense network of nerve fibers that can interact closely with tumor cells to affect tumor growth and provide a pathway for tumor cell dissemination [10,11,12,13]. While the molecular factors that regulate TME-tumor cell interactions in pancreatic cancer are beginning to be elucidated, the physiological factors that regulate this interaction are unclear. Here, we describe accumulating evidence that demonstrate the importance of peripheral sympathetic nervous system (SNS) activation of β-adrenergic receptor signaling in influencing pancreatic cancer progression. We discuss the potential use of β-blockers as a novel treatment strategy to block the adverse effects of SNS signaling, and as an adjuvant therapy to complement existing treatment modalities to improve the quality of life of patients with pancreatic cancer.

## 2. Nerve Fibers Are a Component of the Pancreatic Tumor Microenvironment

The pancreatic microenvironment consists of a broad repertoire of cellular and structural components. This includes glandular epithelial cells in the exocrine acini, that are responsible for the secretion of digestive enzymes, and the endocrine Islet of Langerhans that are involved in hormone secretion [14]. Due to its central role in enzyme and hormone production, the pancreas also contains a dense network of blood vessels to allow for efficient, systemic release of its products into circulation. Tumors can arise from different regions within the pancreas. However, they typically arise from the exocrine compartment which accounts for more than 95% of pancreatic cancers [15]. During the development of a tumor, there is an influx of immune cells into the pancreatic TME, including macrophages that are recruited by tumor cells to promote immunosuppression, tumor vascularization, and metastasis [16]. In addition to these common stromal components, the pancreatic TME also contains several unique components that may contribute to the aggressive nature of pancreatic cancer [17]. The presence of myofibroblast-like PSCs have been shown to support the progression of pancreatic cancer [5,6,9,18] while the particularly high levels of hyaluronic acid, a component of extracellular matrix, has also been demonstrated to impede the delivery of therapeutic agents [19]. Additionally, the extensive network of nerve fibers from the autonomic nervous system forms an integral part of the unique architecture of the pancreatic microenvironment and may also influence cancer progression [20,21]. These nerve fibers include the sympathetic splanchnic nerves, vagus nerve and sensory nerve fibers [22]. Under normal physiological conditions, these nerve fibers work together to regulate digestive enzyme secretion and endocrine hormone secretion [22,23,24,25,26]*.* In particular, activation of fibers of the peripheral SNS, which innervate many areas of the pancreas including areas of exocrine and endocrine tissue as well as blood vessels [21], has been shown to regulate endocrine hormone secretion [25,26] and pancreatic norepinephrine content [27]. Pancreatic tumors are often associated with hyperinnervation, which occurs early in hyperplasia before the transition into overt, malignant disease [28], and is often linked to elevated levels of neuroplasticity markers in the pancreatic TME [11,29,30]. Despite their normal physiological functions, these nerve fibers can also serve as an alternative route for the dissemination of tumor cells, whereby tumor cell invasion into nerve fibers (perineural invasion) [10,12,13,31] is associated with neuropathic pain, a common characteristic of pancreatic cancer [12].

**Figure 1:**
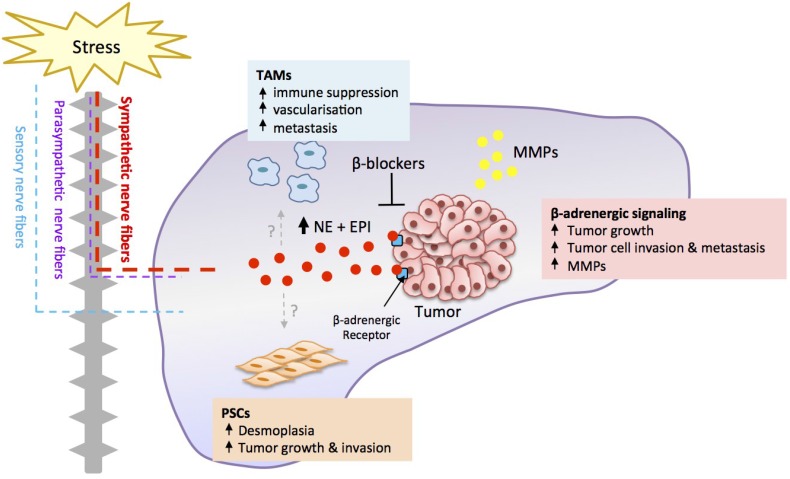
Neural regulation of pancreatic cancer.

Physiological activation of the SNS results in the release of the neurotransmitter norepinephrine from the postganglionic nerve fiber terminus [32] into the pancreas which leads to the elevation of intrapancreatic norepinephrine content [27]. Norepinephrine release from nerve fibers and the adrenal medulla may also be induced by nicotine via activation of nicotinic acetylcholine receptors [33]. Both tumor cells and pancreatic stromal cells express SNS-responsive adrenergic receptors suggesting that SNS signaling could potentially impact the progression of pancreatic cancer (Figure 1). Autocrine response to neurotransmitters is also plausible as norepinephine and epinephrine are synthesized and released by pancreatic duct epithelial cells and pancreatic cancer cells [34,35]. Although the relationship between stress and clinical cancer progression remains unclear, a meta-analysis of over 126 studies spanning 10 different cancer types showed that stress-related psychosocial factors such as depression and stress-prone personality were associated with higher cancer incidence and poor cancer survival [36]. While pancreatic cancer was not specifically analyzed in this study, a similar association between stress and pancreatic cancer progression is possible, suggested by evidence that that shows relative levels of distress, depression and anxiety to be highest amongst patients with pancreatic cancer [37,38,39].

Stress-induced SNS activation elevates intra-pancreatic catecholamine levels (norepinephrine, NE; epinephrine, EPI), which can bind β-adrenergic receptors present on tumor cells to promote tumor cell proliferation and invasion. Stress-induced β-adrenergic signaling may also have effects on various stromal cells present in the pancreatic tumor microenvironment, such as tumor-associated macrophages (TAMs) and pancreatic stellate cells (PSCs) to enhance their tumor supporting functions. These effects collectively result in increased primary tumor growth, tumor cell dissemination and metastasis to distant organs. The adverse effects of stress signaling can be targeted through the use of β-blockers. MMP: Matrix metalloproteinases.

## 3. Orthotopic Preclinical Models Recapitulate Tumor-Stromal Interactions

The significant contribution of the TME to pancreatic cancer progression described above highlights the importance of using preclinical disease models that faithfully recapitulate tumor-stromal interactions. The significant impact of adrenergic signaling on pancreatic cancer cell behavior (proliferation [40,41], motility [40,42] and invasion [40,42,43]) has been studied *in vitro*. A few pre-clinical studies have investigated the effect of stress on pancreatic cancer progression *in vivo*, however these were conducted using subcutaneous models that do not replicate the complexity of the pancreatic TME [44,45]. Therefore, in order to fully recapitulate the comprehensive effects of stress-induced adrenergic signaling on the pancreatic TME and the possible effect this may have on cancer progression, the use of orthotopic cancer models is needed.

There is considerable evidence that tumors grown ectopically behave differently to their orthotopic counterparts. Comparative microarray analysis of a panel of human pancreatic cancer cell lines revealed that gene expression differed greatly when tumor cells were grown *in vitro* compared to *in vivo,* or when tumor cells were implanted ectopically into subcutaneous tissue compared to orthotopically into the pancreas, demonstrating the importance of the organ microenvironment in affecting tumor cell gene expression [46]. In addition, co-implantation studies revealed the importance of stromal cells in modulating tumor growth [47]. Co-implantation of tumor cells with organ-specific stromal cells into ectopic sites significantly enhanced vascular development and affected the kinetics of tumor growth compared to tumor cells injected alone, further highlighting the impact of the stromal environment in regulating tumor progression [47]. This is supported by numerous studies that demonstrated the role of an orthotopic organ microenvironment in conferring distinct growth and therapeutic response profiles [48,49,50,51,52]. Subcutaneous tumors exhibited greater sensitivity to treatment than orthotopic tumors obtained from the same tumor cell line [52,53]. In contrast to orthotopic preclinical models, ectopic mouse models of cancer rarely result in metastasis, as demonstrated in studies of colon cancer [54], gallbladder carcinoma [55], prostate cancer [56,57], renal cell carcinoma [58] and pancreatic cancer [59]. These differences in cancer progression between orthotopic and ectopic models may be attributed to the lack of appropriate stromal cells to drive metastasis, such as pancreatic stellate cells which in the context of pancreatic cancer have been shown to aid in tumor cell invasion and metastasis [5,6]. Collectively, these studies emphasize the importance of studying pancreatic cancer in its natural, or orthotopic, environment to ensure the clinical relevance of findings and to allow for rapid translation.

Several orthotopic mouse models of pancreatic cancer are well established. These involve either the injection of tumor cell suspensions [59,60,61] or surgically transplanting pancreatic tumor fragments into the pancreas [62,63]. However, prior to the development of *in vivo* imaging technologies, non-invasive monitoring of primary tumor growth and dissemination in these models was challenging until the late stages of disease, therefore restricting the development of diagnostics and the development of treatment options to the very late stages of pancreatic cancer when disease had already progressed significantly. As a result, non-orthotopic models using subcutaneous injection of pancreatic cancer cells into the flank were often used to allow for easy monitoring of tumor growth. Recent advances in imaging technology using ultrasound and fluorescence now allow for non-invasive assessment of tumor growth in the pancreas [64]. Furthermore, optical bioluminescence imaging of tumor cells that are transduced to express Firefly or *Renilla* luciferase has also emerged as a highly sensitive alternative to fluorescence and ultrasound imaging to allow real-time monitoring of pancreatic tumor development and metastasis [6,65]. In order to recapitulate more accurately the tumor-stromal interactions that are present in the tumors of patients, patient-derived xenograft (PDX) models—where small tumor fragments from excised patient tumor samples are engrafted in immune-deficient mice—are now increasingly used in the studies of various cancer types [66,67,68,69,70]. These PDX models using either ectopic or orthotopic tumors have been shown to be genetically stable over several *in vivo* passages and have been used for the identification of biomarkers to predict therapeutic response [71] and the evaluation of combination therapy efficacy [72].

## 4. Stress Regulates Pancreatic Cancer Progression 

These various lines of evidence—that stress-induced SNS signaling promotes the progression of a number of cancers [73,74,75,76], and that the pancreatic microenvironment is responsive to SNS signaling—spurred a series of pre-clinical studies to investigate whether chronic stress promotes pancreatic cancer progression in the orthotopic setting. In order to examine the impact of chronic stress on pancreatic cancer progression in tumors grown in the appropriate stroma, we used a mouse model of pancreatic cancer whereby Panc-1 tumor cells were implanted into the tail of the pancreas of nude mice [65]. To physiologically activate SNS signaling pathways, mice were subjected to repeated restraint stress and longitudinal bioluminescence imaging was used to track the effect of stress on cancer progression [77]. Repeated restraint is a frequently used experimental method that increases levels of stress neurotransmitters and hormones [73,74,78]. Chronic stress significantly increased primary tumor growth, an effect that was detectable 21 days after tumor cell implantation and eventually resulted in >10-fold increase in tumor size 42 days post tumor cell implantation. This was associated with a significant increase in primary tumor mass in stressed mice at the conclusion of the experiment. Interestingly, such stress-enhanced growth of the primary tumor was not observed in previous studies using orthotopic models of breast cancer [73], which suggests the sensitivity of primary tumor growth to chronic stress may differ between cancer types.

To date, surgical resection of the primary tumor remains the main curative treatment option for pancreatic cancer. In order to recapitulate standard clinical treatment, we resected the orthotopic pancreatic tumor and used bioluminescence imaging to ensure that tumor-free margins (R0) were obtained [77]. At the time of resection, we examined the adjacent pancreas for disseminated disease. Bioluminescence imaging revealed that chronic stress also induced local dissemination of tumor cells into adjacent, normal, pancreatic tissue. In the clinical context, it is plausible that these cells may seed recurrence if not removed by total pancreatectomy. Additional histological analyses will be required to determine if these cells were present in the pancreatic parenchyma or were transiting through blood or lymphatic vessels. To the extent that chronic stress drives dissemination of tumor cells in patients with pancreatic cancer, these observations provide a possible explanation for the high levels of tumor recurrence in the clinical setting, even after successful R0 resection of the primary tumor. Consistent with the concept that stress drives tumor cell dissemination, stress also increased distant metastasis to liver, spleen and lymph nodes within the gastrointestinal tract compared to control mice [77]. Similar stress-enhanced metastasis was observed in our earlier studies using an orthotopic model of breast cancer [73] and in mouse models of ovarian cancer [74], suggesting metastatic and invasive behavior of tumor cells across multiple cancer types are sensitive to stress signaling. This is of particular significance in pancreatic cancer as local recurrence and distant metastasis are common in patients, even after potential curative resection of the primary tumor [79,80]. Our findings suggest that stress signaling could potentially drive local recurrence of pancreatic cancer and distant metastasis, leading to poor outcomes for patients.

The effects of stress on pancreatic cancer progression are not unique to the restraint paradigm, but have been observed using other experimental situations that activate the conserved stress response. Exposure to sound stress activated neural signaling as indicated by elevated systemic epinephrine and enlarged adrenal glands, and increased pancreatic tumor volume in an ectopic pancreatic cancer model [44]. Another social stress paradigm using twice weekly changes in cage composition similarly promoted pancreatic tumor growth [45]. Chronic stress also drives the progression of other cancer types including breast cancer [73], prostate cancer [75], ovarian cancer [74,78,81], and acute lymphoblastic leukemia [76], highlighting the generalizability of stress effects on cancer progression. These data show that chronic stress increases the growth and dissemination of pancreatic cancer and suggest that sensitivity to stress signaling may be a general phenomenon that impacts many tumor types. Collectively, these findings also suggest that inhibiting stress signaling may be a novel strategy to slow disease progression and improve the condition of patients with pancreatic cancer.

## 5. Stress Acts through a β-Adrenergic Signaling Pathway

Mechanistic intervention studies have characterized a key role for β-adrenergic receptor signaling in the effects of stress on pancreatic cancer progression. Stress-induced activation of the SNS leads to the release of catecholaminergic neurotransmitters, norepinephrine and epinephrine, systemically from the adrenal glands or locally from postganglionic sympathetic nerve fiber termini within the pancreatic microenvironment. These catecholamines can then bind to α- and β-adrenergic receptors to elicit cellular effects [32]. Human pancreatic cancer cell lines including Panc-1, BxPC-3, HDPE6-C7, MIA Paca-2 and PC-2, and stromal cells found in the pancreas including macrophages, fibroblasts and endothelial cells express β-adrenergic receptors [41,45,82,83,84,85,86,87,88,89,90]. We showed that treatment of mice with the non-selective β-antagonist, propranolol, reduced the effect of stress on primary tumor growth and tumor cell dissemination into the adjacent normal pancreas [77]. To minimize the induction of stress by daily subcutaneous injection and handling, these studies used subcutaneously implanted osmotic minipumps for continuous drug administration. The findings indicate that β-adrenergic receptor signaling is necessary for the effects of chronic stress on pancreatic cancer and provide evidence that β-blockade may be used to pharmacologically block the adverse effects of stress.

Consistent with a key role for β-adrenergic signaling in pancreatic cancer progression, *in vitro* and *in vivo* studies have shown that activation of β-adrenergic receptor signaling is sufficient to drive pancreatic cancer progression in ectopic models. Activation of β-adrenergic receptors by isoproterenol, a non-selective β-adrenergic receptor agonist, promoted pancreatic cancer cell proliferation *in vitro* [83,91] and treatment with isoproterenol significantly promoted tumor growth in an ectopic mouse model of pancreatic cancer [86]. To examine if β-adrenergic receptor signaling is sufficient to drive pancreatic cancer progression in the context of the pancreatic stroma, we treated mice bearing orthotopic pancreatic tumors with isoproterenol during the period of tumor development. Isoproterenol significantly increased primary tumor mass and increased dissemination of tumor cells into the adjacent pancreas and to distant tissues. These findings revealed that β-adrenergic signaling alone was sufficient to mimic the effect of stress on pancreatic cancer progression [77]. Other studies similarly documented the critical role of β-adrenergic signaling in driving the progression of other cancer types [73,74,76,92,93].

## 6. β-Adrenergic Signaling Drives Tumor Cell Invasion

Tumor cell invasion plays a key role in pancreatic cancer recurrence and metastasis [79,80,94]. We therefore sought to determine if the effects of β-adrenergic signaling on cancer invasion within the pancreatic microenvironment are due to direct effects on tumor cells or through the effects of β-adrenergic signaling to the pancreatic microenvironment. A number of studies have shown that *in vitro* activation of β-adrenergic receptors promotes the invasiveness of numerous human pancreatic cancer cell lines including Panc-1, MIA Paca-2 and BxPC-3 [40,43,91]. Consistent with those studies, we found that treatment with isoproterenol increased basement membrane invasion by Panc-1 pancreatic cells in a dose-dependent manner, and that these effects were blocked by the β-blocker propranolol.

It is well established that matrix metalloproteinases (MMPs) facilitate tumor cell invasion by degrading the surrounding extracellular matrix [95,96,97]. Previous studies demonstrated that stress was associated with increased *MMP* expression in tumors of mice [73,74] and in patient tumor samples [98]. Therefore, it is plausible that β-adrenergic signaling mediates stress effects on pancreatic cancer cell invasion and metastasis by upregulating *MMP* expression either by tumor cells or by stromal cells in the pancreatic TME. Quantitation of transcript levels in cultured tumor cells demonstrated that isoproterenol upregulated *MMP2* and *MMP9* expression by two-fold and four-fold, respectively [77], with the endogenous catecholamine norepinephrine shown to have similar effects [43]. To confirm the effects of stress-enhanced *MMP* expression *in vivo*, we further analyzed the effect of chronic restraint stress on *MMP* expression in tumor and stromal cells in primary orthotopic pancreatic tumors. The impact of stress on the expression of both *MMP2* and *MMP9* in tumors *in vivo* was far greater than that observed *in vitro*. Stress selectively increased *MMP9* expression in tumor cells (~50-fold compared to non-stressed mice) while stress enhanced *MMP2* expression in cells from the stromal compartment (>100-fold compared to non-stressed mice) [77]. These findings indicate that the effects of SNS signaling during stress are not limited to pancreatic cancer cells alone, but that β-adrenergic regulation of the pancreatic TME may be influential in driving cancer invasion and distant metastasis (Figure 1). Consistent with these findings from orthotopic mouse models, tumor samples from patients with pancreatic ductal adenocarcinoma have also shown differential MMP expression in the tumor and stromal cell compartments. Histological analyses found that *MMP2* expression was elevated in pancreatic stroma compared to neoplastic epithelium [99]. It was also found that MMP2 activity increased with pancreatic tumor grade [100], highlighting the important role of MMP2 in the progression of pancreatic cancer. Although it is essential to characterize the effect of stress on MMP expression and tumor cell invasion in clinical samples, this line of evidence suggests that stress-induced β-adrenergic signaling may enhance tumor cell dissemination by also affecting stromal cell components within the TME to facilitate tumor cell invasion.

It will also be important to determine which components of the pancreatic TME are responsible for modulating stress-enhanced protease production. One candidate is tumor-associated macrophages which express β-adrenergic receptors [101] and modulate gene expression in response to β-adrenergic signaling [73,98,102]. Given that tumor-associated macrophages induce immunosuppression, promote metastasis and impair response to treatment [103,104], it is plausible that stress either promotes macrophage infiltration into pancreatic tumors or changes the phenotype of tumor-associated macrophages to mediate stress-enhanced effects on MMP expression and cancer progression. Consistent with this hypothesis, our earlier studies of breast cancer have demonstrated that stress promotes macrophage recruitment to primary tumors, which was necessary for stress-enhanced metastasis [73]. Another stromal cell type that has been implicated in the progression of pancreatic cancer is PSCs, which have been described as a source of MMPs and other pro-metastatic molecules [105]. PSCs promote desmoplasia, a characteristic feature in pancreatic cancers, by increasing the production of extracellular matrix molecules including collagen and fibronectin [8,9]*.* Interactions between PSCs and tumor cells invoke a positive feedback loop to enhance the proliferation of either cell type and induce regional metastasis of pancreatic cancer cells in mice [5,9]. Additionally, PSCs can disseminate together with pancreatic cancer cells to distant organs [106], suggesting PSCs may also assist to establish metastatic outgrowths in distant organs. The effect of stress signaling on PSCs remains unknown. PSCs are similar to hepatic stellate cells in their morphology and function [107,108], suggesting that PSCs may be similarly responsive to NE-enhanced proliferation as observed for hepatic stellate cells [109]. Considering the pivotal role of PSCs in pancreatic cancer progression, it is essential to examine the impact of stress on PSCs themselves, and on tumor cell-PSC interactions in the effects on pancreatic cancer progression. Together, these findings suggest that regulation of tumor-stromal interactions to elevate protease production may be one mechanism by which stress promotes the dissemination of pancreatic cancer cells.

## 7. Stress Regulates Tumor Cell Proliferation and Apoptosis

In addition to its effects on tumor cell invasion, stress may also drive pancreatic tumor growth by increasing the rate of tumor cell proliferation. Endogenous stress neurotransmitters were demonstrated to promote pancreatic cancer cell proliferation *in vitro* [40,41] via the activation of β-adrenergic signaling [83,86,91]. Several signaling pathways downstream of β-adrenergic receptors have been proposed to mediate stress-enhanced tumor cell proliferation, including activation of HIF-1α-dependent transcription [44] and activation of the ERK/MAPK pathway [86]. It is also noteworthy that stress-enhanced tumor growth was not limited to the orthotopic model [77], but was similarly observed in ectopic models of pancreatic cancer [45,86], suggesting that stress may act directly on pancreatic cancer cells to modulate cell proliferation.

In addition to promoting tumor cell proliferation, stress may promote tumor growth by inhibiting apoptosis of tumor cells. Several *in vitro* studies have shown that activation of β-adrenergic signaling pathways protects tumor cells against cell death [78,110,111]. Additionally, inhibiting β-adrenergic signaling to pancreatic cancer cells induced apoptosis by suppressing the Ras/Akt/NFκB signaling pathway [41,85]. Similar findings were also reported in studies of hemangioma [112], neuroblastoma [113], melanoma [114], and gastric cancer [115]. However, the effectiveness of β-blockade in inducing apoptosis was inconsistent in ectopic mouse models of pancreatic cancer [44,86], which may be attributed to cell-intrinsic differences in β-blocker sensitivity in the pancreatic cancer cell lines used. Further investigation to clarify the effect of β-blockers in different pancreatic cell lines, and in the context of the pancreatic TME using orthotopic models and samples from pancreatic patients who previously used β-blockers (e.g., for co-morbid hypertension) will provide additional insights into the effect of stress signaling on tumor cell proliferation and apoptosis.

## 8. Translation of β-Blockers for the Treatment of Pancreatic Cancer

To effectively translate β-blockers for the treatment of pancreatic cancer, it will be essential to determine the treatment regimen and to define which patients will most benefit. β-blockers, commonly used for the treatment of hypertension in the clinical setting, may also be used in the adjuvant setting to improve the efficacy of existing chemotherapy regimens or in patients with unresectable tumors. For patients with advanced stage pancreatic cancer, an unresectable tumor often restricts the choice of treatment to chemotherapy. Gemcitabine is at the core of the standard-of-care chemotherapy regimen for pancreatic cancer and results in an average 5-8 months prolongation of survival [116,117,118,119]. Recent trials of several combination therapies for pancreatic cancer including gemcitabine combined to the epidermal growth factor receptor (EGFR) antagonist erlotinib [120], FOLFIRINOX (a combination of oxaliplatin, irinotecan, leucovorin and flurouracil) [121], or gemcitabine combined with the albumin-bound taxane derivative nab-paclitaxel [122] have shown improvement of overall survival from weeks to months. However, these strategies have been accompanied by increased side effects and toxicity such as peripheral neuropathy. Therefore, combination therapies with better efficacy and safety profiles are urgently needed for the treatment of pancreatic cancer.

The preclinical studies presented here suggest that β-blockers could be used to complement existing chemotherapeutic strategies to improve cancer outcome in patients with pancreatic cancer [44,77,86]. While a small retrospective study did not support this concept [123], positive results were obtained in a recent prospective trial [124]. The study was a randomized, open-label, single-center clinical trial to compare the effectiveness of propranolol plus etodolac, a COX-2 inhibitor, in combination with gemcitabine plus *nab-*paclitaxel [124]. Addition of the β-blocker to the treatment strategy significantly improved the median overall survival by 7.7 months (9.3 months in chemotherapy arm, *n* = 17, *vs.* 17 months in chemotherapy + β-blocker, *n* = 20). Furthermore, β-blockade also reduced treatment complications including weight loss and peripheral neuropathy. While further studies are required in order to conclusively demonstrate the beneficial effects of β-blockers on clinical cancer outcome, these findings support the feasibility of using β-blockers to complement current chemotherapeutic agents to improve overall survival. As they are safe and well characterized drugs, β-blockers may have additional benefits of reducing side effects without the risk of increased toxicity.

Several epidemiological studies have shown the use of β-blockers at the time of cancer diagnosis to be associated with improved cancer outcome in a number of solid cancer types such as breast cancer [125,126,127,128], prostate cancer [129], melanoma [130,131], ovarian cancer [132] or non-small-cell lung cancer [133]. In addition to targeting the adverse effects of chronic stress, β-blockers may be useful to counter diagnosis-related anxiety and surgical stress that accompanies tumor resection. Stress is a frequent consequence of cancer diagnosis, particularly in the case of pancreatic cancer where patients are often faced with an extremely poor prognosis [37,38,39]. As pancreatic cancer cells and stromal cells express β-adrenergic receptors [41,45,82,83,84,85,86], it is therefore plausible that the use of β-blockers could potentially improve pancreatic cancer outcome by attenuating the effects of stress via the inhibition of SNS signaling.

An additional window of opportunity to block stress signaling may be during surgical resection of pancreatic tumors. The perioperative period is a window of time when patients are particularly vulnerable to physiological stress signaling, and accumulating experimental and clinical evidence suggests that surgical stress may drive long-term cancer recurrence [134]. In addition to complementing existing chemotherapeutic agents, β-blockers could be given to pancreatic cancer patients prior to surgical resection to reduce the effect of elevated neurotransmitter levels and limit inflammation with the goal of improving cancer outcomes. It is possible that perioperative β-blockade may additionally modulate the microenvironment of metastatic targets to limit metastatic outgrowth of circulating tumor cells as a result of surgery.

## 9. Conclusions

Increasing evidence indicates that stress signaling drives pancreatic cancer progression [44,45,77], with tumor cell invasion being particularly sensitive to the adverse effects of stress [77]. As stress levels are often high in pancreatic cancer patients [37,38,39] and metastatic dissemination is largely responsible for cancer mortality [79,80], intervening in stress signaling may benefit pancreatic cancer patients. Identification of β-adrenergic receptor signaling pathways as key regulators of the effects of stress on pancreatic cancer progression suggests that β-blockers may be repurposed from their current role as a cardiac medication to complement existing anti-cancer therapeutic strategies. Translation of these preclinical findings will be guided by studies that clarify which β-blockers optimally target the pancreatic tumor microenvironment and that characterize their mechanism of action. Translation will be additionally aided by stratifying patient sub-populations who will optimally benefit from stress-reducing interventions.

## References

[1] Quail D.F., Joyce J.A. (2013). Microenvironmental regulation of tumor progression and metastasis. Nat Med..

[2] Klemm F., Joyce J.A. (2015). Microenvironmental regulation of therapeutic response in cancer. Trends Cell Biol..

[3] Provenzano P.P., Hingorani S.R. (2013). Hyaluronan, fluid pressure, and stromal resistance in pancreas cancer. Br. J. Cancer.

[4] Oettle H. (2014). Progress in the knowledge and treatment of advanced pancreatic cancer: From benchside to bedside. Cancer Treat. Rev..

[5] Vonlaufen A., Joshi S., Qu C., Phillips P.A., Xu Z., Parker N.R., Toi C.S., Pirola R.C., Wilson J.S., Goldstein D. (2008). Pancreatic stellate cells: Partners in crime with pancreatic cancer cells. Cancer Res..

[6] Hwang R.F., Moore T., Arumugam T., Ramachandran V., Amos K.D., Rivera A., Ji B., Evans D.B., Logsdon C.D. (2008). Cancer-associated stromal fibroblasts promote pancreatic tumor progression. Cancer Res..

[7] Siegel R., Ma J., Zou Z., Jemal A. (2014). Cancer statistics, 2014. CA Cancer J. Clin..

[8] Apte M.V., Park S., Phillips P.A., Santucci N., Goldstein D., Kumar R.K., Ramm G.A., Buchler M., Friess H., McCarroll J.A. (2004). Desmoplastic reaction in pancreatic cancer: Role of pancreatic stellate cells. Pancreas.

[9] Bachem M.G., Schunemann M., Ramadani M., Siech M., Beger H., Buck A., Zhou S., Schmid-Kotsas A., Adler G. (2005). Pancreatic carcinoma cells induce fibrosis by stimulating proliferation and matrix synthesis of stellate cells. Gastroenterology.

[10] Hirai I., Kimura W., Ozawa K., Kudo S., Suto K., Kuzu H., Fuse A. (2002). Perineural invasion in pancreatic cancer. Pancreas.

[11] Ma J., Jiang Y., Jiang Y., Sun Y., Zhao X. (2008). Expression of nerve growth factor and tyrosine kinase receptor a and correlation with perineural invasion in pancreatic cancer. J. Gastroenterol. Hepatol..

[12] Ceyhan G.O., Bergmann F., Kadihasanoglu M., Altintas B., Demir I.E., Hinz U., Muller M.W., Giese T., Buchler M.W., Giese N.A. (2009). Pancreatic neuropathy and neuropathic pain—A comprehensive pathomorphological study of 546 cases. Gastroenterology.

[13] Pour P.M., Bell R.H., Batra S.K. (2003). Neural invasion in the staging of pancreatic cancer. Pancreas.

[14] Ellis H. (2013). Anatomy of the pancreas and spleen. Surgery.

[15] Bond-Smith G., Banga N., Hammond T.M., Imber C.J. (2012). Pancreatic adenocarcinoma. BMJ.

[16] Mielgo A., Schmid M.C. (2013). Impact of tumour associated macrophages in pancreatic cancer. BMB Rep..

[17] Feig C., Gopinathan A., Neesse A., Chan D.S., Cook N., Tuveson D.A. (2012). The pancreas cancer microenvironment. Clin. Cancer Res..

[18] Schneiderhan W., Diaz F., Fundel M., Zhou S., Siech M., Hasel C., Moller P., Gschwend J.E., Seufferlein T., Gress T. (2007). Pancreatic stellate cells are an important source of mmp-2 in human pancreatic cancer and accelerate tumor progression in a murine xenograft model and cam assay. J. Cell Sci..

[19] Provenzano P.P., Cuevas C., Chang A.E., Goel V.K., Von Hoff D.D., Hingorani S.R. (2012). Enzymatic targeting of the stroma ablates physical barriers to treatment of pancreatic ductal adenocarcinoma. Cancer Cell.

[20] Tang S.C., Peng S.J., Chien H.J. (2014). Imaging of the islet neural network. Diabetes Obes. Metab..

[21] Rodriguez-Diaz R., Abdulreda M.H., Formoso A.L., Gans I., Ricordi C., Berggren P.O., Caicedo A. (2011). Innervation patterns of autonomic axons in the human endocrine pancreas. Cell Metab..

[22] Bockman D.E. (2007). Nerves in the pancreas: What are they for?. Am. J. Surg..

[23] Teff K.L. (2008). Visceral nerves: Vagal and sympathetic innervation. JPEN J. Parenter. Enteral. Nutr..

[24] Holmgren S., Olsson C. (2011). Autonomic control of glands and secretion: A comparative view. Auton. Neurosci..

[25] Ahren B., Ericson L.E., Lundquist I., Loren I., Sundler F. (1981). Adrenergic innervation of pancreatic islets and modulation of insulin secretion by the sympatho-adrenal system. Cell Tissue Res..

[26] Kurose T., Seino Y., Nishi S., Tsuji K., Taminato T., Tsuda K., Imura H. (1990). Mechanism of sympathetic neural regulation of insulin, somatostatin, and glucagon secretion. Am. J. Physiol..

[27] Hisatomi A., Maruyama H., Orci L., Vasko M., Unger R.H. (1985). Adrenergically mediated intrapancreatic control of the glucagon response to glucopenia in the isolated rat pancreas. J. Clin. Investig..

[28] Stopczynski R.E., Normolle D.P., Hartman D.J., Ying H., DeBerry J.J., Bielefeldt K., Rhim A.D., DePinho R.A., Albers K.M., Davis B.M. (2014). Neuroplastic changes occur early in the development of pancreatic ductal adenocarcinoma. Cancer Res..

[29] Ceyhan G.O., Giese N.A., Erkan M., Kerscher A.G., Wente M.N., Giese T., Buchler M.W., Friess H. (2006). The neurotrophic factor artemin promotes pancreatic cancer invasion. Ann. Surg..

[30] Ceyhan G.O., Schafer K.H., Kerscher A.G., Rauch U., Demir I.E., Kadihasanoglu M., Bohm C., Muller M.W., Buchler M.W., Giese N.A. (2010). Nerve growth factor and artemin are paracrine mediators of pancreatic neuropathy in pancreatic adenocarcinoma. Ann. Surg..

[31] Liu B., Lu K.Y. (2002). Neural invasion in pancreatic carcinoma. Hepatobiliary Pancreat. Dis. Int..

[32] Weiner H. (1992). Perturbing the Organism: The Biology of Stressful Experience.

[33] Haass M., Kubler W. (1997). Nicotine and sympathetic neurotransmission. Cardiovasc. Drugs Ther..

[34] Al-Wadei M.H., Al-Wadei H.A., Schuller H.M. (2012). Pancreatic cancer cells and normal pancreatic duct epithelial cells express an autocrine catecholamine loop that is activated by nicotinic acetylcholine receptors alpha3, alpha5, and alpha7. Mol. Cancer Res..

[35] Al-Wadei M.H., Al-Wadei H.A., Schuller H.M. (2012). Effects of chronic nicotine on the autocrine regulation of pancreatic cancer cells and pancreatic duct epithelial cells by stimulatory and inhibitory neurotransmitters. Carcinogenesis.

[36] Chida Y., Hamer M., Wardle J., Steptoe A. (2008). Do stress-related psychosocial factors contribute to cancer incidence and survival?. Nat. Clin. Pract. Oncol..

[37] Clark K.L., Loscalzo M., Trask P.C., Zabora J., Philip E.J. (2010). Psychological distress in patients with pancreatic cancer—An understudied group. Psychooncology.

[38] Zabora J., BrintzenhofeSzoc K., Curbow B., Hooker C., Piantadosi S. (2001). The prevalence of psychological distress by cancer site. Psychooncology.

[39] Carlson L.E., Angen M., Cullum J., Goodey E., Koopmans J., Lamont L., MacRae J.H., Martin M., Pelletier G., Robinson J. (2004). High levels of untreated distress and fatigue in cancer patients. Br. J. Cancer.

[40] Huang X.Y., Wang H.C., Yuan Z., Huang J., Zheng Q. (2012). Norepinephrine stimulates pancreatic cancer cell proliferation, migration and invasion via beta-adrenergic receptor-dependent activation of p38/mapk pathway. Hepato-Gastroenterology.

[41] Zhang D., Ma Q., Shen S., Hu H. (2009). Inhibition of pancreatic cancer cell proliferation by propranolol occurs through apoptosis induction: The study of beta-adrenoceptor antagonist's anticancer effect in pancreatic cancer cell. Pancreas.

[42] Guo K., Ma Q., Li J., Wang Z., Shan T., Li W., Xu Q., Xie K. (2013). Interaction of the sympathetic nerve with pancreatic cancer cells promotes perineural invasion through the activation of stat3 signaling. Mol. Cancer Ther..

[43] Guo K., Ma Q.Y., Wang L.C., Hu H.T., Li J.H., Zhang D., Zhang M. (2009). Norepinephrine-induced invasion by pancreatic cancer cells is inhibited by propranolol. Oncol. Rep..

[44] Shan T., Ma J., Ma Q., Guo K., Guo J., Li X., Li W., Liu J., Huang C., Wang F. (2013). Beta2-ar-hif-1alpha: A novel regulatory axis for stress-induced pancreatic tumor growth and angiogenesis. Curr. Mol. Med..

[45] Schuller H.M., Al-Wadei H.A., Ullah M.F., Plummer H.K. (2012). Regulation of pancreatic cancer by neuropsychological stress responses: A novel target for intervention. Carcinogenesis.

[46] Nakamura T., Fidler I.J., Coombes K.R. (2007). Gene expression profile of metastatic human pancreatic cancer cells depends on the organ microenvironment. Cancer Res..

[47] Borgstrom P., Oh P., Czarny M., Racine B., Schnitzer J.E. (2013). Co-implanting orthotopic tissue creates stroma microenvironment enhancing growth and angiogenesis of multiple tumors. F1000 Res..

[48] Wilmanns C., Fan D., O’Brian C.A., Bucana C.D., Fidler I.J. (1992). Orthotopic and ectopic organ environments differentially influence the sensitivity of murine colon carcinoma cells to doxorubicin and 5-fluorouracil. Int. J. Cancer.

[49] Dong Z., Radinsky R., Fan D., Tsan R., Bucana C.D., Wilmanns C., Fidler I.J. (1994). Organ-specific modulation of steady-state mdr gene expression and drug resistance in murine colon cancer cells. J. Natl. Cancer Inst..

[50] Graves E.E., Vilalta M., Cecic I.K., Erler J.T., Tran P.T., Felsher D., Sayles L., Sweet-Cordero A., Le Q.T., Giaccia A.J. (2010). Hypoxia in models of lung cancer: Implications for targeted therapeutics. Clin. Cancer Res..

[51] Ho K.S., Poon P.C., Owen S.C., Shoichet M.S. (2012). Blood vessel hyperpermeability and pathophysiology in human tumour xenograft models of breast cancer: A comparison of ectopic and orthotopic tumours. BMC Cancer.

[52] Westwood J.A., Potdevin Hunnam T.C., Pegram H.J., Hicks R.J., Darcy P.K., Kershaw M.H. (2014). Routes of delivery for cpg and anti-cd137 for the treatment of orthotopic kidney tumors in mice. PLoS ONE.

[53] Graves E.E., Maity A., Le Q.T. (2010). The tumor microenvironment in non-small-cell lung cancer. Semin. Radiat. Oncol..

[54] Nakajima M., Morikawa K., Fabra A., Bucana C.D., Fidler I.J. (1990). Influence of organ environment on extracellular matrix degradative activity and metastasis of human colon carcinoma cells. J. Natl. Cancer Inst..

[55] Du Q., Jiang L., Wang X.Q., Pan W., She F.F., Chen Y.L. (2014). Establishment of and comparison between orthotopic xenograft and subcutaneous xenograft models of gallbladder carcinoma. Asian Pac. J. Cancer Prev..

[56] Stephenson R.A., Dinney C.P., Gohji K., Ordonez N.G., Killion J.J., Fidler I.J. (1992). Metastatic model for human prostate cancer using orthotopic implantation in nude mice. J. Natl. Cancer Inst..

[57] Greene G.F., Kitadai Y., Pettaway C.A., von Eschenbach A.C., Bucana C.D., Fidler I.J. (1997). Correlation of metastasis-related gene expression with metastatic potential in human prostate carcinoma cells implanted in nude mice using an *in situ* messenger rna hybridization technique. Am. J. Pathol..

[58] Singh R.K., Bucana C.D., Gutman M., Fan D., Wilson M.R., Fidler I.J. (1994). Organ site-dependent expression of basic fibroblast growth factor in human renal cell carcinoma cells. Am. J. Pathol..

[59] Tan M.H., Chu T.M. (1985). Characterization of the tumorigenic and metastatic properties of a human pancreatic tumor-cell line (aspc-1) implanted orthotopically into nude-mice. Tumour Biol..

[60] Bruns C.J., Harbison M.T., Kuniyasu H., Eue I., Fidler I.J. (1999). In vivo selection and characterization of metastatic variants from human pancreatic adenocarcinoma by using orthotopic implantation in nude mice. Neoplasia.

[61] Marincola F.M., Drucker B.J., Siao D.Y., Hough K.L., Holder W.D. (1989). The nude mouse as a model for the study of human pancreatic cancer. J. Surg. Res..

[62] Fu X., Guadagni F., Hoffman R.M. (1992). A metastatic nude-mouse model of human pancreatic cancer constructed orthotopically with histologically intact patient specimens. Proc. Natl. Acad. Sci. USA.

[63] Bouvet M., Yang M., Nardin S., Wang X., Jiang P., Baranov E., Moossa A.R., Hoffman R.M. (2000). Chronologically-specific metastatic targeting of human pancreatic tumors in orthotopic models. Clin. Exp. Metastasis.

[64] Snyder C.S., Kaushal S., Kono Y., Cao H.S., Hoffman R.M., Bouvet M. (2009). Complementarity of ultrasound and fluorescence imaging in an orthotopic mouse model of pancreatic cancer. BMC Cancer.

[65] Chai M.G., Kim-Fuchs C., Angst E., Sloan E.K. (2013). Bioluminescent orthotopic model of pancreatic cancer progression. J. Vis. Exp..

[66] DeRose Y.S., Wang G., Lin Y.C., Bernard P.S., Buys S.S., Ebbert M.T., Factor R., Matsen C., Milash B.A., Nelson E. (2011). Tumor grafts derived from women with breast cancer authentically reflect tumor pathology, growth, metastasis and disease outcomes. Nat. Med..

[67] Merk J., Rolff J., Becker M., Leschber G., Fichtner I. (2009). Patient-derived xenografts of non-small-cell lung cancer: A pre-clinical model to evaluate adjuvant chemotherapy?. Eur. J. Cardiothorac. Surg..

[68] Oh B.Y., Lee W.Y., Jung S., Hong H.K., Nam D.H., Park Y.A., Huh J.W., Yun S.H., Kim H.C., Chun H.K. (2015). Correlation between tumor engraftment in patient-derived xenograft models and clinical outcomes in colorectal cancer patients. Oncotarget.

[69] Gupta J., Igea A., Papaioannou M., Lopez-Casas P.P., Llonch E., Hidalgo M., Gorgoulis V.G., Nebreda A.R. (2015). Pharmacological inhibition of p38 mapk reduces tumor growth in patient-derived xenografts from colon tumors. Oncotarget.

[70] Schuller A.G., Barry E.R., Jones R.D., Henry R.E., Frigault M.M., Beran G., Linsenmayer D., Hattersley M., Smith A., Wilson J. (2015). The met inhibitor azd6094 (savolitinib, hmpl-504) induces regression in papillary renal cell carcinoma patient-derived xenograft models. Clin. Cancer Res..

[71] Rubio-Viqueira B., Jimeno A., Cusatis G., Zhang X., Iacobuzio-Donahue C., Karikari C., Shi C., Danenberg K., Danenberg P.V., Kuramochi H. (2006). An *in vivo* platform for translational drug development in pancreatic cancer. Clin. Cancer Res..

[72] Walters D.M., Lindberg J.M., Adair S.J., Newhook T.E., Cowan C.R., Stokes J.B., Borgman C.A., Stelow E.B., Lowrey B.T., Chopivsky M.E. (2013). Inhibition of the growth of patient-derived pancreatic cancer xenografts with the mek inhibitor trametinib is augmented by combined treatment with the epidermal growth factor receptor/her2 inhibitor lapatinib. Neoplasia.

[73] Sloan E.K., Priceman S.J., Cox B.F., Yu S., Pimentel M.A., Tangkanangnukul V., Arevalo J.M., Morizono K., Karanikolas B.D., Wu L. (2010). The sympathetic nervous system induces a metastatic switch in primary breast cancer. Cancer Res..

[74] Thaker P.H., Han L.Y., Kamat A.A., Arevalo J.M., Takahashi R., Lu C., Jennings N.B., Armaiz-Pena G., Bankson J.A., Ravoori M. (2006). Chronic stress promotes tumor growth and angiogenesis in a mouse model of ovarian carcinoma. Nat. Med..

[75] Hassan S., Karpova Y., Baiz D., Yancey D., Pullikuth A., Flores A., Register T., Cline J.M., D’Agostino R., Danial N. (2013). Behavioral stress accelerates prostate cancer development in mice. J. Clin. Investig..

[76] Lamkin D.M., Sloan E.K., Patel A.J., Chiang B.S., Pimentel M.A., Ma J.C., Arevalo J.M., Morizono K., Cole S.W. (2012). Chronic stress enhances progression of acute lymphoblastic leukemia via beta-adrenergic signaling. Brain Behav. Immun..

[77] Kim-Fuchs C., Le C.P., Pimentel M.A., Shackleford D., Ferrari D., Angst E., Hollande F., Sloan E.K. (2014). Chronic stress accelerates pancreatic cancer growth and invasion: A critical role for beta-adrenergic signaling in the pancreatic microenvironment. Brain Behav. Immun..

[78] Sood A.K., Armaiz-Pena G.N., Halder J., Nick A.M., Stone R.L., Hu W., Carroll A.R., Spannuth W.A., Deavers M.T., Allen J.K. (2010). Adrenergic modulation of focal adhesion kinase protects human ovarian cancer cells from anoikis. J. Clin. Investig..

[79] Hishinuma S., Ogata Y., Tomikawa M., Ozawa I., Hirabayashi K., Igarashi S. (2006). Patterns of recurrence after curative resection of pancreatic cancer, based on autopsy findings. J. Gastrointest. Surg..

[80] Kleeff J., Reiser C., Hinz U., Bachmann J., Debus J., Jaeger D., Friess H., Buchler M.W. (2007). Surgery for recurrent pancreatic ductal adenocarcinoma. Ann. Surg..

[81] Armaiz-Pena G.N., Gonzalez-Villasana V., Nagaraja A.S., Rodriguez-Aguayo C., Sadaoui N.C., Stone R.L., Matsuo K., Dalton H.J., Previs R.A., Jennings N.B. (2015). Adrenergic regulation of monocyte chemotactic protein 1 leads to enhanced macrophage recruitment and ovarian carcinoma growth. Oncotarget.

[82] Weddle D.L., Tithoff P., Williams M., Schuller H.M. (2001). Beta-adrenergic growth regulation of human cancer cell lines derived from pancreatic ductal carcinomas. Carcinogenesis.

[83] Askari M.D., Tsao M.S., Schuller H.M. (2005). The tobacco-specific carcinogen, 4-(methylnitrosamino)-1-(3-pyridyl)-1-butanone stimulates proliferation of immortalized human pancreatic duct epithelia through beta-adrenergic transactivation of egf receptors. J. Cancer Res. Clin. Oncol..

[84] Zhang D., Ma Q.Y., Hu H.T., Zhang M. (2010). Beta2-adrenergic antagonists suppress pancreatic cancer cell invasion by inhibiting creb, nfkappab and ap-1. Cancer Biol. Ther..

[85] Zhang D., Ma Q., Wang Z., Zhang M., Guo K., Wang F., Wu E. (2011). Beta2-adrenoceptor blockage induces g1/s phase arrest and apoptosis in pancreatic cancer cells via ras/akt/nfkappab pathway. Mol. Cancer.

[86] Lin X.P., Luo K., Lv Z.W., Huang J. (2012). Beta-adrenoceptor action on pancreatic cancer cell proliferation and tumor growth in mice. Hepato-Gastroenterology.

[87] Kondo H., Takeuchi S., Togari A. (2013). Beta-adrenergic signaling stimulates osteoclastogenesis via reactive oxygen species. Am. J. Physiol. Endocrinol. Metab..

[88] Stiles J., Amaya C., Pham R., Rowntree R.K., Lacaze M., Mulne A., Bischoff J., Kokta V., Boucheron L.E., Mitchell D.C. (2012). Propranolol treatment of infantile hemangioma endothelial cells: A molecular analysis. Exp. Ther. Med..

[89] Turner N.A., Porter K.E., Smith W.H., White H.L., Ball S.G., Balmforth A.J. (2003). Chronic beta2-adrenergic receptor stimulation increases proliferation of human cardiac fibroblasts via an autocrine mechanism. Cardiovasc. Res..

[90] Lamyel F., Warnken-Uhlich M., Seemann W.K., Mohr K., Kostenis E., Ahmedat A.S., Smit M., Gosens R., Meurs H., Miller-Larsson A. (2011). The beta2-subtype of adrenoceptors mediates inhibition of pro-fibrotic events in human lung fibroblasts. Naunyn Schmied. Arch. Pharmacol..

[91] Schuller H.M., Al-Wadei H.A., Majidi M. (2008). Gaba b receptor is a novel drug target for pancreatic cancer. Cancer.

[92] Magnon C., Hall S.J., Lin J., Xue X., Gerber L., Freedland S.J., Frenette P.S. (2013). Autonomic nerve development contributes to prostate cancer progression. Science.

[93] Campbell J.P., Karolak M.R., Ma Y., Perrien D.S., Masood-Campbell S.K., Penner N.L., Munoz S.A., Zijlstra A., Yang X., Sterling J.A. (2012). Stimulation of host bone marrow stromal cells by sympathetic nerves promotes breast cancer bone metastasis in mice. PLoS Biol..

[94] Steg A.D., Bevis K.S., Katre A.A., Ziebarth A., Dobbin Z.C., Alvarez R.D., Zhang K., Conner M., Landen C.N. (2012). Stem cell pathways contribute to clinical chemoresistance in ovarian cancer. Clin. Cancer Res..

[95] Bloomston M., Zervos E.E., Rosemurgy A.S. (2002). Matrix metalloproteinases and their role in pancreatic cancer: A review of preclinical studies and clinical trials. Ann. Surg. Oncol..

[96] Egeblad M., Werb Z. (2002). New functions for the matrix metalloproteinases in cancer progression. Nat. Rev. Cancer.

[97] Kessenbrock K., Plaks V., Werb Z. (2010). Matrix metalloproteinases: Regulators of the tumor microenvironment. Cell.

[98] Lutgendorf S.K., Lamkin D.M., Jennings N.B., Arevalo J.M., Penedo F., DeGeest K., Langley R.R., Lucci J.A., Cole S.W., Lubaroff D.M. (2008). Biobehavioral influences on matrix metalloproteinase expression in ovarian carcinoma. Clin. Cancer Res..

[99] Maatta M., Soini Y., Liakka A., Autio-Harmainen H. (2000). Differential expression of matrix metalloproteinase (mmp)-2, mmp-9, and membrane type 1-mmp in hepatocellular and pancreatic adenocarcinoma: Implications for tumor progression and clinical prognosis. Clin. Cancer Res..

[100] Durlik M., Gardian K. (2012). Metalloproteinase 2 and 9 activity in the development of pancreatic cancer. Pol. Prz. Chir..

[101] Izeboud C.A., Mocking J.A., Monshouwer M., van Miert A.S., Witkamp R.F. (1999). Participation of beta-adrenergic receptors on macrophages in modulation of lps-induced cytokine release. J. Recept. Signal Transduct. Res..

[102] Hasko G., Nemeth Z.H., Szabo C., Zsilla G., Salzman A.L., Vizi E.S. (1998). Isoproterenol inhibits il-10, tnf-alpha, and nitric oxide production in raw 264.7 macrophages. Brain Res. Bull..

[103] Mitchem J.B., Brennan D.J., Knolhoff B.L., Belt B.A., Zhu Y., Sanford D.E., Belaygorod L., Carpenter D., Collins L., Piwnica-Worms D. (2013). Targeting tumor-infiltrating macrophages decreases tumor-initiating cells, relieves immunosuppression, and improves chemotherapeutic responses. Cancer Res..

[104] Campbell A.S., Albo D., Kimsey T.F., White S.L., Wang T.N. (2005). Macrophage inflammatory protein-3alpha promotes pancreatic cancer cell invasion. J. Surg. Res..

[105] Phillips P.A., McCarroll J.A., Park S., Wu M.J., Pirola R., Korsten M., Wilson J.S., Apte M.V. (2003). Rat pancreatic stellate cells secrete matrix metalloproteinases: Implications for extracellular matrix turnover. Gut.

[106] Xu Z., Vonlaufen A., Phillips P.A., Fiala-Beer E., Zhang X., Yang L., Biankin A.V., Goldstein D., Pirola R.C., Wilson J.S. (2010). Role of pancreatic stellate cells in pancreatic cancer metastasis. Am. J. Pathol..

[107] Apte M.V., Haber P.S., Applegate T.L., Norton I.D., McCaughan G.W., Korsten M.A., Pirola R.C., Wilson J.S. (1998). Periacinar stellate shaped cells in rat pancreas: Identification, isolation, and culture. Gut.

[108] Buchholz M., Kestler H.A., Holzmann K., Ellenrieder V., Schneiderhan W., Siech M., Adler G., Bachem M.G., Gress T.M. (2005). Transcriptome analysis of human hepatic and pancreatic stellate cells: Organ-specific variations of a common transcriptional phenotype. J. Mol. Med. (Berl.).

[109] Oben J.A., Yang S., Lin H., Ono M., Diehl A.M. (2003). Norepinephrine and neuropeptide y promote proliferation and collagen gene expression of hepatic myofibroblastic stellate cells. Biochem. Biophys. Res. Commun..

[110] Sastry K.S., Karpova Y., Prokopovich S., Smith A.J., Essau B., Gersappe A., Carson J.P., Weber M.J., Register T.C., Chen Y.Q. (2007). Epinephrine protects cancer cells from apoptosis via activation of camp-dependent protein kinase and bad phosphorylation. J. Biol. Chem..

[111] Eng J.W., Reed C.B., Kokolus K.M., Pitoniak R., Utley A., Bucsek M.J., Ma W.W., Repasky E.A., Hylander B.L. (2015). Housing temperature-induced stress drives therapeutic resistance in murine tumour models through beta2-adrenergic receptor activation. Nat. Commun..

[112] Ji Y., Li K., Xiao X., Zheng S., Xu T., Chen S. (2012). Effects of propranolol on the proliferation and apoptosis of hemangioma-derived endothelial cells. J. Pediatr. Surg..

[113] Wolter J.K., Wolter N.E., Blanch A., Partridge T., Cheng L., Morgenstern D.A., Podkowa M., Kaplan D.R., Irwin M.S. (2014). Anti-tumor activity of the beta-adrenergic receptor antagonist propranolol in neuroblastoma. Oncotarget.

[114] Wrobel L.J., Le Gal F.A. (2015). Inhibition of human melanoma growth by a non-cardioselective beta-blocker. J. Investig. Dermatol..

[115] Liao X., Che X., Zhao W., Zhang D., Bi T., Wang G. (2010). The beta-adrenoceptor antagonist, propranolol, induces human gastric cancer cell apoptosis and cell cycle arrest via inhibiting nuclear factor kappab signaling. Oncol. Rep..

[116] Storniolo A.M., Enas N.H., Brown C.A., Voi M., Rothenberg M.L., Schilsky R. (1999). An investigational new drug treatment program for patients with gemcitabine: Results for over 3000 patients with pancreatic carcinoma. Cancer.

[117] Rougier P., Riess H., Manges R., Karasek P., Humblet Y., Barone C., Santoro A., Assadourian S., Hatteville L., Philip P.A. (2013). Randomised, placebo-controlled, double-blind, parallel-group phase III study evaluating aflibercept in patients receiving first-line treatment with gemcitabine for metastatic pancreatic cancer. Eur. J. Cancer.

[118] Herrmann R., Bodoky G., Ruhstaller T., Glimelius B., Bajetta E., Schuller J., Saletti P., Bauer J., Figer A., Pestalozzi B. (2007). Gemcitabine plus capecitabine compared with gemcitabine alone in advanced pancreatic cancer: A randomized, multicenter, phase iii trial of the swiss group for clinical cancer research and the central european cooperative oncology group. J. Clin. Oncol..

[119] Burris H., Storniolo A.M. (1997). Assessing clinical benefit in the treatment of pancreas cancer: Gemcitabine compared to 5-fluorouracil. Eur. J. Cancer.

[120] Zhou R.Y., Shanas R., Nelson M.A., Bhattacharyya A., Shi J.Q. (2010). Increased expression of the heterogeneous nuclear ribonucleoprotein k in pancreatic cancer and its association with the mutant p53. Int. J. Cancer.

[121] Conroy T., Desseigne F., Ychou M., Bouche O., Guimbaud R., Becouarn Y., Adenis A., Raoul J.L., Gourgou-Bourgade S., de la Fouchardiere C. (2011). Folfirinox *versus* gemcitabine for metastatic pancreatic cancer. N. Engl. J. Med..

[122] Von Hoff D.D., Ervin T., Arena F.P., Chiorean E.G., Infante J., Moore M., Seay T., Tjulandin S.A., Ma W.W., Saleh M.N. (2013). Increased survival in pancreatic cancer with nab-paclitaxel plus gemcitabine. N. Engl. J. Med..

[123] Shah S.M., Carey I.M., Owen C.G., Harris T., Dewilde S., Cook D.G. (2011). Does beta-adrenoceptor blocker therapy improve cancer survival? Findings from a population-based retrospective cohort study. Br. J. Clin. Pharmacol..

[124] Bhattacharyya G.S., Babu K.G., Bondarde S.A., Biswas G., Ranade A., Parikh P.M., Bascomb N.F., Malhotra H. (2015). Effect of coadministered beta blocker and cox-2 inhibitor to patients with pancreatic cancer prior to receiving albumin-bound (nab) paclitaxel. J. Clin. Oncol..

[125] Powe D.G., Voss M.J., Zanker K.S., Habashy H.O., Green A.R., Ellis I.O., Entschladen F. (2010). Beta-blocker drug therapy reduces secondary cancer formation in breast cancer and improves cancer specific survival. Oncotarget.

[126] Melhem-Bertrandt A., Chavez-Macgregor M., Lei X., Brown E.N., Lee R.T., Meric-Bernstam F., Sood A.K., Conzen S.D., Hortobagyi G.N., Gonzalez-Angulo A.M. (2011). Beta-blocker use is associated with improved relapse-free survival in patients with triple-negative breast cancer. J. Clin. Oncol..

[127] Botteri E., Munzone E., Rotmensz N., Cipolla C., de Giorgi V., Santillo B., Zanelotti A., Adamoli L., Colleoni M., Viale G. (2013). Therapeutic effect of beta-blockers in triple-negative breast cancer postmenopausal women. Breast Cancer Res. Treat..

[128] Barron T.I., Connolly R.M., Sharp L., Bennett K., Visvanathan K. (2011). Beta blockers and breast cancer mortality: A population- based study. J. Clin. Oncol..

[129] Grytli H.H., Fagerland M.W., Fossa S.D., Tasken K.A., Haheim L.L. (2013). Use of beta-blockers is associated with prostate cancer-specific survival in prostate cancer patients on androgen deprivation therapy. Prostate.

[130] De Giorgi V., Grazzini M., Gandini S., Benemei S., Lotti T., Marchionni N., Geppetti P. (2011). Treatment with beta-blockers and reduced disease progression in patients with thick melanoma. Arch. Int. Med..

[131] Lemeshow S., Sorensen H.T., Phillips G., Yang E.V., Antonsen S., Riis A.H., Lesinski G.B., Jackson R., Glaser R. (2011). Beta-blockers and survival among danish patients with malignant melanoma: A population-based cohort study. Cancer Epidemiol. Biomark. Prev..

[132] Diaz E.S., Karlan B.Y., Li A.J. (2012). Impact of beta blockers on epithelial ovarian cancer survival. Gynecol. Oncol..

[133] Wang H.M., Liao Z.X., Komaki R., Welsh J.W., O'Reilly M.S., Chang J.Y., Zhuang Y., Levy L.B., Lu C., Gomez D.R. (2013). Improved survival outcomes with the incidental use of beta-blockers among patients with non-small-cell lung cancer treated with definitive radiation therapy. Ann. Oncol..

[134] Horowitz M., Neeman E., Sharon E., Ben-Eliyahu S. (2015). Exploiting the critical perioperative period to improve long-term cancer outcomes. Nat. Rev. Clin. Oncol..

